# Robot-assisted laparoscopic treatment of appendicovesical fistula in a child: a case report and literature review

**DOI:** 10.3389/fped.2025.1610586

**Published:** 2025-08-05

**Authors:** Zheng Fang, Yi Sun, Zhongxu Wang, Xiangming Yan, Ting Zhang, Xu Cao, Tianyi Wang, Hongchao Wang, Jun Liu, Ting Feng, Shu Dai

**Affiliations:** ^1^ Department of Urology, Children’s Hospital of Soochow University, Suzhou, China; ^2^ Department of Ultrasound, Children’s Hospital of Soochow University, Suzhou, China

**Keywords:** pediatric, appendicovesical fistula, robot-assisted laparoscopic surgery, contrast-enhanced ultrasound, case report

## Abstract

Appendicovesical fistula (AVF) is a rare condition that occurs in children characterized by an abnormal communication between the appendix and the bladder, caused by diseases such as inflammation or malignancy. Due to the lack of specific clinical symptoms and the difficulty in confirming the diagnosis with traditional imaging examinations such as ultrasound and CT, the disease often has a protracted course. This paper reports a case of a pediatric case of AVF presenting with dysuria, urinary frequency, and diarrhea. The diagnosis was confirmed by contrast-enhanced ultrasound (CEUS), and the patient successfully underwent robot-assisted laparoscopic partial cystectomy and appendectomy without postoperative complications. This case demonstrates an innovative application of CEUS combined with robot-assisted laparoscopic surgery for the diagnosis and treatment of a pediatric case of AVF. In addition, we conducted a literature review on AVF in children.

## Introduction

AVF is a rare pediatric condition with only a few case reports documented worldwide (1). The atypical symptoms and the difficulty in confirming the diagnosis with traditional imaging examinations such as ultrasound and CT often lead to delayed diagnosis, sometimes not until adulthood. Therefore, the diagnosis and reporting of AVF in children are even rarer. Previous cases were mostly confirmed by laparotomy, with preoperative diagnosis being challenging and the prolonged disease course often leading to severe pelvic adhesions, increasing the difficulty of laparoscopic surgery (3). There are currently no reports of robot-assisted laparoscopic surgery for AVF in children (1–3). This study reports a case of a child diagnosed with AVF, who was diagnosed preoperatively by CEUS and treated with robot-assisted laparoscopic surgery (27). The study also reviews the relevant literature to explore the diagnostic and treatment experience of AVF in children.

## Case presentation

A male child aged 6 years and 11 months presented to our hospital due to “difficulty in urination and diarrhea for 2 weeks.” Two weeks prior, the patient presented with difficulty in urination, dribbling urine, dysuria, urinary frequency, and watery diarrhea without any obvious cause. The local hospital's anti-infection treatment was ineffective, leading to a referral to our hospital. Physical examination revealed suprapubic tenderness but no palpable masses or rebound tenderness. Urinalysis showed proteinuria (++), occult blood (+++), 1,048 red blood cells/μl, and 1,320 white blood cells/μl, with urine bacterial culture indicating *Pseudomonas aeruginosa*. Both ultrasound and CT suggested the presence of bladder stones. The preoperative diagnosis was bladder stones, and the patient underwent transurethral bladder lithotripsy. During the surgery, a 2 cm × 2 cm × 2 cm oval-shaped stone was found, which was brittle and wrapped with a substance resembling dragon fruit seeds. Local bladder mucosa was edematous, and the bladder wall showed no abnormal protrusions or fistula structures. The child was discharged after a 4-day recovery period. Stone composition analysis revealed calcium phosphate stones, which were considered to be infection-induced.

**Figure 1:**
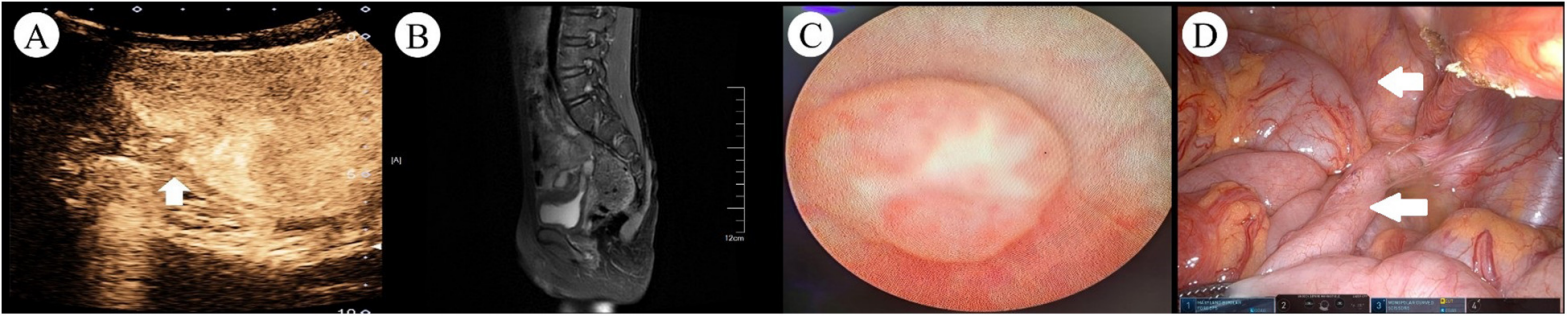
Preoperative imaging and intraoperative findings in a pediatric case of AVF. **(A)** CEUS revealed hyperechoic areas within the intestinal tract and pelvic cavity (arrows indicated the fistula tract). **(B)** Contrast-enhanced MRI demonstrated a small, pointed tubular structure at the superior posterior right aspect of the bladder, with a width of approximately 4 mm. A linear, markedly enhanced signal was observed within the lumen. **(C)** Cystoscopy showed a papillary protrusion on the right lateral wall of the bladder, with no evident fistula opening or foreign material discharge observed at the protrusion site. **(D)** Intraoperative view revealed significant adhesion between the bladder and the appendix in the pelvic cavity, with the appendix appearing swollen (the bladder is marked by the upper indicator, concurrent with appendiceal localization demonstrated by the lower indicator).

Two weeks after discharge, the patient experienced recurrent symptoms of urinary frequency, dysuria, and diarrhea, with urinalysis indicating a urinary tract infection. Ultrasound of the urinary system showed a hypoechoic mass on the right posterior wall of the bladder. After more than 1 month of antibiotic treatment yielded poor efficacy, the patient intermittently exhibited pneumaturia and fecaluria, alongside worsening diarrhea symptoms. Suspicion of a fistula between the intestine and bladder led to readmission. Cystography and barium enema showed no definite fistula imaging. Contrast-enhanced ultrasound (CEUS) after injecting the contrast agent (sulfur hexafluoride microbubbles) into the bladder revealed contrast agent overflowing from the right posterior wall of the bladder into the intestine (Figure 1A). Sacral MRI demonstrated a small tubular shadow at the upper edge of the right posterior side of the bladder, with a strip-like significantly enhanced shadow in the lumen after enhanced scanning, suggestive of a fistula with infection (Figure 1B). The preoperative diagnosis was appendicovesical fistula (AVF).

Cystoscopy and robot-assisted laparoscopic partial cystectomy, appendectomy, and intestinal adhesion lysis were performed. Cystoscopy revealed a papillary protrusion on the right side of the bladder (Figure 1C); however, no obvious fistula opening or foreign body discharge was observed at the protrusion site, prompting withdrawal of the cystoscope. The patient was placed in a supine position. Using the da Vinci surgical system (intra-abdominal pressure 12 mmHg), an 8 mm trocar was inserted through the umbilical incision. The remaining two robotic arm ports were placed according to the principles of dominant hand positioning and robotic surgical channel establishment, with a spacing of 7 cm; a 5 mm auxiliary port was established between the right robotic port and the observation port. The umbilical observation port was connected to the No. 2 robotic arm, and the laparoscope was subsequently inserted into the abdominal cavity. During the surgery, obvious adhesion between the bladder and an enlarged appendix in the pelvic cavity was visible (Figure 1D). Combined with clinical manifestations, AVF was confirmed intraoperatively, and robot-assisted laparoscopic partial cystectomy, appendectomy, and intestinal adhesion lysis were performed.

A urinary catheter was left in place postoperatively, and cefoperazone sodium and sulbactam sodium were used for anti-infection treatment according to the drug sensitivity results. Postoperative pathological examination of the appendix revealed lymphoid hyperplasia changes in the mucosal wall, leading to a diagnosis of chronic appendicitis. The patient recovered well, achieving normal blood tests, urinalysis, and pelvic ultrasound findings by postoperative day 14. Antibiotics were discontinued on the 10th postoperative day, the urinary catheter was removed on the 13th day, and pelvic ultrasound review on the 14th day showed no abscess or effusion, enabling discharge following recovery. The patient was followed up in the outpatient clinic for more than 11 months after surgery, reporting no special discomfort; urinalysis and urinary system ultrasound examinations during this period showed no abnormalities.

## Discussion

The occurrence of AVF in pediatric populations is extremely rare. To date, over 100 cases of AVF across all age groups have been reported in the literature, accounting for 1% to 5% of all enterovesical fistulas, with only 10%–15% of these cases occurring in children (4, 5). James et al. (6) proposed that AVF in children, due to its long and narrow fistulous tract, is often obstructed by feces or stones, presenting a periodic state of obstruction or patency, with extremely atypical symptoms. Some cases remain asymptomatic for years (5), making timely and accurate diagnosis challenging. Our systematic review identified only 17 reported cases of children with AVF from 1976 to 2024 (1–4, 6–15). Including the present case, there are a total of 18 cases, with 14 males and 4 females, and an average age of 8.3 years (range 1–17 years), as shown in Table 1.

**Table 1:** Clinical data of children with AVF.

No.	Author	Year	Gender	Age	Clinical symptoms	Preoperative diagnosis	Diagnostic method	Diagnostic interval	Treatment
1	Present case	2025	Male	6.9 years	Dysuria, pneumaturia, fecaluria, diarrhea (2 months).	AVF	CEUS and MRI	90 days	Robot-assisted lap appendectomy, fistula resection, bladder repair.
2	Yang X et al. (25)	2024	Male	5 years	dysuria, urgency, and frequency (1 week)	AVF	CEUS and cystoscopy	6 months	Lap appendectomy, bladder repair.
3	Li C et al. (15)	2020	Female	17 years	Dysuria, hematuria, fever (20 days).	Pelvic foreign body	Surgical exploration	20 days	Laparotomy: appendectomy, fistula resection, bladder repair, stone removal.
4	Sun B et al. (14)	2017	Male	1 year	Fever, dysuria, fecaluria; history of NEC.	Enterovesical fistula	Cystography and laparoscopic exploration	NA	Lap appendectomy, bladder repair, drainage.
5	Yu k et al. (1)	2016	Male	9.5 years	Urine discharge from abdominal wall incision; cloudy urine.	Urachal cyst	Surgical exploration	NA	Laparotomy: appendectomy, fistula resection, bladder repair.
6	Yu k et al. (1)	2016	Male	7.5 years	Penile pain, hematuria, pneumaturia (1 month).	Bladder tumor	Surgical exploration	1 month	Laparotomy: appendectomy, fistula resection, bladder repair.
7	Trinavarat P et al. (4)	2009	Male	4.7 years	Recurrent UTIs, dysuria, fever, diarrhea (1 month).	AVF	Cystography and cystoscopy	1 year	Laparotomy: appendectomy, fistula resection, bladder repair.
8	Lijuan G et al. (13)	2007	Female	12 years	Dysuria, diarrhea (2 years).	NA	Cystography and surgical exploration	2 years	Laparotomy: appendectomy, fistula resection, stone removal.
9	Gao LJ et al. (13)	2007	Male	8 years	Dysuria, cloudy urine, pneumaturia (6 months); history of appendicitis.	NA	Surgical exploration	6 months	Laparotomy: appendectomy, fistula resection.
10	Hamill J et al. (6)	2006	Male	6 years	Abnormal urine odor, diarrhea; history of abdominal pain.	AVF	Cystography and cystoscopy	18 months	Surgical treatment (details unspecified).
11	Abubakar et al. (12)	2006	Male	1 year	Cessation of flatus, fecaluria, abdominal pain; history of megacolon.	NA	Surgical exploration	1 year	Laparotomy: appendectomy, bladder repair, colostomy.
12	Wang M et al. (1)	2003	Male	17 years	Cloudy urine, dysuria (12 years); history of ascaris expulsion.	NA	Cystoscopy and surgical exploration	12 years	Laparotomy: appendectomy, fistula resection, bladder repair.
13	Steinberg et al. (2)	1999	Male	16 years	Dysuria, pneumaturia, fecaluria; history of Crohn's disease.	Crohn's disease	Surgical exploration	6 months	Laparotomy: appendectomy, fistula repair.
14	Yamamoto et al. (3)	1997	Male	1.7 years	Fever, pyuria (15 days).	NA	Cystoscopy and laparoscopic exploration	15 days	Lap appendectomy, fistula repair via small incision.
15	Cakmak et al. (9)	1997	Female	16 years	Recurrent UTIs, dysuria, abdominal pain; history of cystic fibrosis.	AVF	Cystoscopy and biopsy	1 year	Laparotomy: appendectomy, bladder defect repair.
16	Sheng XF et al. (10)	1997	Male	12 years	Dysuria, penile pain, abdominal distension, diarrhea (5 days); history of appendicitis.	AVF	Diagnosis based on typical history and symptoms	10 days	Laparotomy: appendectomy, bladder repair.
17	Ma J et al. (1)	1993	Male	9 years	Dysuria, pneumaturia, fecaluria (8 years); history of appendicitis.	AVF	Cystography	8 years	Laparotomy: appendectomy, fistula resection, cystostomy.
18	Rizen.B K. et al. (7)	1976	Female	9 years	Fever, abdominal pain, dysuria; history of appendicitis.	NA	Intravenous pyelography and cystoscopy	NA	Laparotomy (details unspecified).

AVF, appendicovesical fistula; UTIs, urinary tract infections; NEC, necrotizing enterocolitis; Lap, laparoscopic, MRI, magnetic resonance imaging; NA, not available.

In adults, the etiology of AVF is diverse, encompassing appendicitis, malignant tumors of the ileocecal region, Crohn's disease, villous adenoma, neurofibroma, appendiceal mucinous cyst, appendiceal diverticulitis, and parasitic infections (16). In contrast, among children, the etiology of AVF is almost invariably due to perforation from appendicitis and subsequent adhesion to the bladder. Among the 17 cases, 16 were caused by appendicitis, 1 was due to surgical misoperation (11). Given that the symptoms of pediatric appendicitis are often atypical and the diagnostic process can be influenced by underlying diseases, leading to delayed diagnosis and eventual development of AVF. Moreover, once the fistula forms, the discharge of appendix contents may alleviate symptoms, further complicating preoperative diagnosis.

In previous literature, underlying diseases in some children (such as Crohn's disease, cystic fibrosis, and congenital megacolon) masked the diagnosis of appendicitis, but postoperative pathology confirmed that AVF was still caused by chronic appendicitis (2, 9, 12). Although the incidence and perforation rate of pediatric appendicitis are higher than in adults, AVF in children is rarer. This may be because the omentum in children is not fully developed, making it less likely to form localized inflammatory masses that adhere to the bladder wall during appendicitis, thereby reducing the probability of AVF formation after abscess rupture (3). 1In addition, in recent years, guidelines have recommended appendectomy as the preferred treatment for appendicitis (17), fundamentally reducing the occurrence of AVF. A review of the literature also shows that patients diagnosed with AVF who had a clear history of appendicitis were primarily documented before 2007, and conservative treatment was often used at that time, reflecting era-specific characteristics.

Children with AVF primarily exhibit urinary system symptoms including recurrent urinary tract infections, difficulty in urination, bladder stones, and abdominal pain, with relatively specific and rare symptoms being pneumaturia, fecaluria, and diarrhea. Among the 17 childs, all exhibited symptoms of urinary tract infection, with 7 cases presenting pneumaturia and fecaluria, 5 cases with diarrhea, and 1 case with Ascaris lumbricoides discharge through the urethra. Pneumaturia and fecaluria are one of the specific manifestations of AVF in children, but they are usually not significant and occur intermittently, which is related to the narrow appendiceal tubular fistulous tract and can be used to differentiate from other types of enterovesical fistulas. Abubakar et al. (12) reported a case of a child with congenital megacolon leading to intestinal obstruction, who subsequently developed fecaluria symptoms. The reason was that the intestinal adhesion was aggravated after appendiceal perforation, leading to increased intestinal pressure, which caused the intestinal contents to enter the bladder through the fistula. In addition, bladder stones accompanied by chronic diarrhea are also one of the specific symptoms of AVF in children. Sheng Xinfu (10) reported a case of a child with urinary retention and diarrhea caused by bladder stones obstructing the urethra, and the diarrhea stopped immediately after the placement of a urinary catheter, which helped to make a preoperative diagnosis of AVF. The child in this case had both bladder stones with diarrhea symptoms and intermittent pneumaturia and fecaluria. Combined with auxiliary examinations, AVF was considered highly likely. Therefore, the diagnosis of AVF in children relies on specific clinical manifestations to infer the existence of an intermittently open enterovesical fistula, and the appendix is the slender structure causing this phenomenon, providing a clear direction for subsequent auxiliary examinations.

In the auxiliary examinations for AVF in children, common diagnostic methods include CT, cystoscopy, cystography, and gastrointestinal tract imaging. Among the 18 childs, 7 were diagnosed with AVF preoperatively, 2 cases utilized CEUS combined with complementary imaging, 1 case diagnosed based on typical symptoms, and the remaining 4 cases diagnosed through cystography, cystoscopy, or a combination of both. Traditionally, CT and cystoscopy have been considered the preferred methods for diagnosing AVF (18). However, CT poses radiation risks to children and often fails to clearly display the fistula orifice or tract; cystoscopy, while capable of detecting larger fistula orifices, is prone to missing smaller ones, and the periodic opening of AVF may result in negative examination findings. Rizen et al. (7) reported a case of a child where the initial cystoscopy revealed purulent discharge from the bladder fistula orifice, confirming the presence of the fistula tract, but a second cystoscopy conducted preoperatively, despite careful examination, failed to detect any signs of the fistula orifice, and intraoperative exploration also did not reveal a bladder fistula orifice.

The present case exhibited similar characteristics, with no abnormalities detected in the bladder wall during the first cystoscopy with holmium laser lithotripsy, but a small papillary protrusion on the right side of the bladder wall was observed during a second cystoscopy prior to robot-assisted laparoscopic surgery. It is evident that cystoscopy is prone to missing smaller bladder fistula orifices, and the periodic opening of AVF may lead to negative examination results. Cystography is also commonly used for diagnosis, but due to the viscosity of the contrast agent and the narrow appendiceal lumen, the results are often negative. Therefore, most cases require intraoperative confirmation. The present case utilized CEUS and MRI, which dynamically observed the shape of the fistula tract, achieving a high diagnostic accuracy rate. Compared with traditional cystography, CEUS uses sulfur hexafluoride microbubble solution, which is non-radioactive, more easily passes through narrow lumens, and offers higher diagnostic sensitivity and accuracy (19–24). Therefore, CEUS combined with MRI is the preferred preoperative diagnostic option for AVF in children. Recent studies further validate the multimodal utility of CEUS (25). Beyond dynamically visualizing bladder-appendiceal fistula morphology, intravesical contrast instillation enables functional colonic imaging, successfully capturing the complete pathway of contrast agent migration from the appendix into the ascending colon. This radiation-free 3D imaging capability makes it particularly suitable for children requiring repeated examinations.

The current preferred treatment for AVF in children is surgical resection of the appendix, partial bladder, and fistula tract on the basis of sensitive antibiotic use (26). Before 2016, laparotomy was commonly used. In 1997, Yamamoto et al. (3) first reported the use of laparoscopic-assisted exploration, which, despite being converted to open surgery due to equipment limitations at the time, provided a new approach for AVF treatment. Of the 17 AVF in children cases reported in the literature, only 2 were successfully treated with laparoscopy, possibly because these cases had long diagnostic times, with a median diagnosis time of 365 days (10–4,380 days), and the prolonged disease course led to severe pelvic adhesions, increasing the difficulty of minimally invasive surgery, hence the continued frequent use of laparotomy. The present case, with a diagnosis time of 90 days, underwent robot-assisted laparoscopic surgery after preoperative assessment, including partial cystectomy, appendectomy, and intestinal adhesion lysis. The child recovered well postoperatively, without complications, was discharged after recovery, and had complete symptom relief during outpatient follow-up. We opted for robot-assisted laparoscopic surgery due to its widespread application in pediatric urology. This approach provides flexible robotic arm maneuverability, enhanced visual information, and stable surgical quality, particularly in the confined pelvic space (27). Studies have shown that robot-assisted bladder surgery is superior to traditional surgery in terms of surgical time, postoperative hospital stay, and therapeutic effect, indicating that its application in the pelvis is safe and effective (28–30). Therefore, for AVF in children with a short disease course and preoperative diagnosis, minimally invasive surgery, including laparoscopic surgery or robot-assisted laparoscopic surgery, is recommended.

In summary, AVF in children is extremely rare, with appendicitis being the predominant underlying cause, although it often initially presents as a urinary tract infection. Preoperative diagnosis is challenging, and intermittent fecaluria, pneumaturia, and bladder stones accompanied by diarrhea are relatively specific clinical manifestations. For the auxiliary examination of AVF in children, CEUS and MRI should be the first choices. For AVF in children diagnosed preoperatively, laparoscopic or robot-assisted laparoscopic surgery can achieve good therapeutic effects.

## Data Availability

The original contributions presented in the study are included in the article/Supplementary Material, further inquiries can be directed to the corresponding author.
